# Numerical tests of magnetoreception models assisted with behavioral experiments on American cockroaches

**DOI:** 10.1038/s41598-021-91815-x

**Published:** 2021-06-09

**Authors:** Kai Sheng Lee, Rainer Dumke, Tomasz Paterek

**Affiliations:** 1 School of Physical and Mathematical Sciences, Nanyang Technological University, Singapore, 637371 Singapore; 2 Centre for Quantum Technologies, National University of Singapore, Singapore, 117543 Singapore; 3 Institute of Theoretical Physics and Astrophysics, Faculty of Mathematics, Physics, and Informatics, University of Gdańsk, 80-308 Gdańsk, Poland

**Keywords:** Biophysics, Biological physics

## Abstract

Many animals display sensitivity to external magnetic field, but it is only in the simplest organisms that the sensing mechanism is understood. Here we report on behavioural experiments where American cockroaches (*Periplaneta americana*) were subjected to periodically rotated external magnetic fields with a period of 10 min. The insects show increased activity when placed in a periodically rotated Earth-strength field, whereas this effect is diminished in a twelve times stronger periodically rotated field. We analyse established models of magnetoreception, the magnetite model and the radical pair model, in light of this adaptation result. A broad class of magnetite models, based on single-domain particles found in insects and assumption that better alignment of magnetic grains towards the external field yields better sensing and higher insect activity, is shown to be excluded by the measured data. The radical-pair model explains the data if we assume that contrast in the chemical yield on the order of one in a thousand is perceivable by the animal, and that there also exists a threshold value for detection, attained in an Earth-strength field but not in the stronger field.

## Introduction

The Earth’s magnetic field has existed for at least 3.5 billion years, the result of an electrically conducting fluid core^1^. Most of life has evolved in the presence of the Earth’s field and it is unsurprising that organisms have developed adaptations taking advantage of it. For instance, magnetotactic bacteria are observed to passively align with magnetic field lines, a phenomena coined as magnetotaxis^2^. This is achieved via chains of magnetic crystals encased in membranes rigidly connected to the bacteria so that the magnetic torque can directly steer the whole organism. For larger animals, an added layer of complexity in translating magnetic information to biologically useful neuronal signals is required. We refer to this process of sensing and translating as magnetoreception or magnetic sensing (we do not require that it acts as a compass, i.e. provides directional information).

A plethora of species across the animal phyla, from insects like planthoppers^3^ or honeybees^4–6^, fish like yellowfin tuna^7^ or sockeye salmon^8^, birds like homing pigeons^9^ and mammals like bats^10,11^, have been observed to exhibit the ability of magnetoreception. A fuller compilation of known species can be found in^12^. However, beyond magnetotactic bacteria, the mechanism behind magnetoreception is not known. The usual candidate explanations include the magnetite model^13^ and the radical pair model^14–16^. The former involves the presence of ferromagnetic deposits that act like tiny compasses, while the latter involves chemical reactions with distinct products that can be modulated by an external magnetic field. It is suggested that both methods might be present in a single animal^12^. Alternative ideas exist, for example, many experimental findings are compatible with the model based on magneto-elastic properties of cells^17^, where the magnetic information is directly transduced into electrical signals without necessitating a magnetic torque. Clearly, more data is required to narrow down theoretical possibilities and ultimately localise relevant receptor organs and sensory pathways^18^.

A major practical significance of magnetoreception is not only the ability to sense the Earth field of approximately 0.5 G (Gauss) but also sensitivity to time-varying fields with amplitudes in the range 10–100 $\upmu$G, observed in European robins^19^ and American cockroaches^20^. This is achieved by biological sensors at room temperature and compact sizes, and once understood will lead to robust man-made magnetic sensors.

Here we report on experiments with American cockroaches (*Periplaneta americana*) that confirm their magnetoreception and show adaptation of the sensory mechanism to the Earth’s magnetic field. We then use these results to put constraints on the magnetite and radical pair models. Cockroaches are good candidates for studies on magnetic sensing for several reasons. Their genome has been completely sequenced^21^, opening the way towards controlled and focused genetic studies related to magnetoreception. The size of cockroaches makes them easy to handle and translates to compact table top experiments. Cockroaches were shown to be magnetisable, with very long magnetisation decay ranging from about an hour in a living insect to about 2 days in a dead one^22^. Finally, cockroaches have already been observed to react to changes in external magnetic fields^20,23,24^, and it is known that the sensing involves the protein cryptochrome^25^. All these behavioural experiments have been conducted in the group of Dr. Martin Vacha in Masaryk University, and the added value of the present work is an independent confirmation of magnetoreception in *Periplaneta americana*.

In summary, we video recorded cockroaches in various magnetic fields and utilised tracking software to extract the time when they were active (rotating or translating). Cockroaches are found to be more active in a periodically rotated Earth-strength magnetic field. When instead they were faced with a periodically rotated magnetic field of 5 G, 12 times the strength of the geomagnetic field, cockroach activity was diminished. We simulate leading explanations of magnetoreception and conclude that the magnetite model, with the sizes of single-domain grains extracted from insects, cannot explain our data if it follows assumption that better alignment of magnetic moments to external field translates to higher activity of the animal. The radical-pair model is compatible with measured data if we impose that contrast in the chemical yield on the order of one in a thousand is perceived by the animal, and that there also exists a minimum yield for detection, attained in an Earth strength field but not in the stronger 5 G field.

## Results

We first describe our methodology, inspired by that of the group of Dr. Martin Vacha in Masaryk University and extending Refs.^20,23–25^, and then present obtained results.

**Figure 1:**
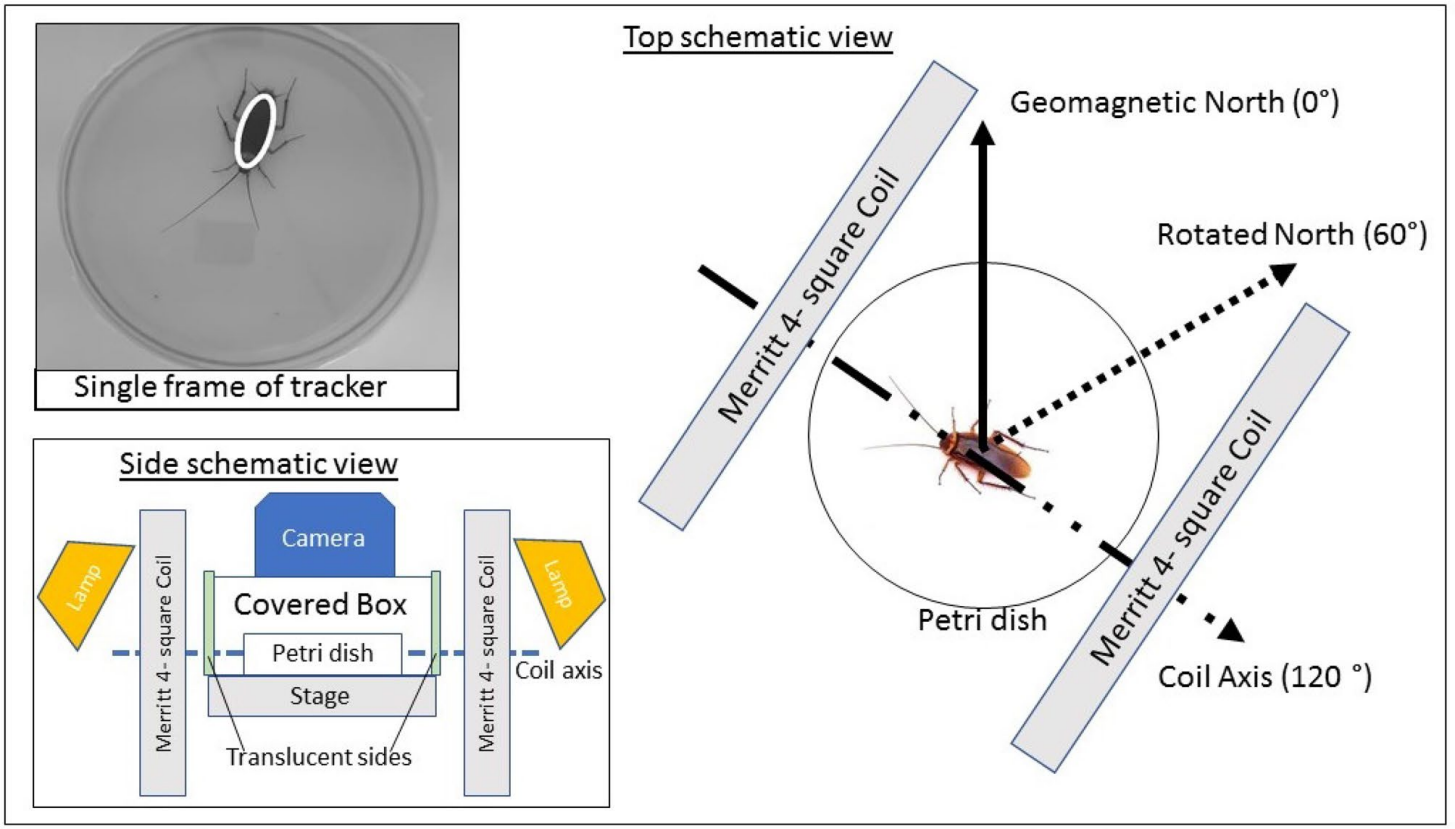
Schematic of experimental setup (not to scale). Cockroaches were placed in Petri dishes and levelled inside the Merritt coil used to rotate the geomagnetic field by $60^\circ \pm 5^\circ$ clockwise (preserving inclination angle, i.e. only horizontal component of the geomagnetic field is modified). The coil alternates between being switched on and off every 5 min, from 6 am to 6 pm. A camera records cockroach motion from the top of a covered box prepared to minimise visual cues from surrounding (bottom left inset). Tracking software recognises the insect (top left inset showing ellipse fitted to the cockroach) and stores its location and angle in the horizontal plane. Our figure of merit is the activity time defined as the time a cockroach spends translating or rotating.

The schematic of our setup is depicted in Fig. 1. Experimental procedure and data processing is described in detail in “Methods” section. In essence, the cockroaches were video recorded in an isolated room by a fully automated apparatus. A camera was attached on top of the box that contained a single cockroach in a Petri dish at a time. Visual cues from the surrounding were minimised. The box was installed inside the Merritt coil, a configuration of four square loops of wires that produced the uniform magnetic field across the area accessible to the cockroach.

Randomised permutations of experimental conditions and cockroach specimen were made. Repeated cockroaches were used, with the constraint that the same cockroach is never used consecutively. The exact sequence can be found in the text files available as Additional Information. Three experimental conditions were studied: one control and two different classes of tests. In the control runs (N = 29), the coils were switched off at all times. In the first class of tests (N = 29), which we will refer to as the Rotated Earth Strength Field (RESF), the coil was arranged such that, when turned on, its field rotates the magnetic north by $60 \pm 5 ^\circ$ clockwise in the horizontal plane (see Fig. 1). The magnitude and inclination angle of the rotated field was unchanged with respect to the geomagnetic field. The coil was then periodically switched on and off every 5 min, from 6 am to 6 pm. In the second class of tests, referred to as 5 G tests (N = 16), a different Merritt coil was used to produce a field with the horizontal component rotated 60 $\pm 5^\circ$ clockwise and increased to 5 G (so that the total field has magnitude about 5.01 G), which is about 12 times stronger than the Earth’s magnetic field (see Supplementary Information for illustration). The same switching profile was used as in the first class of tests.

**Figure 2:**
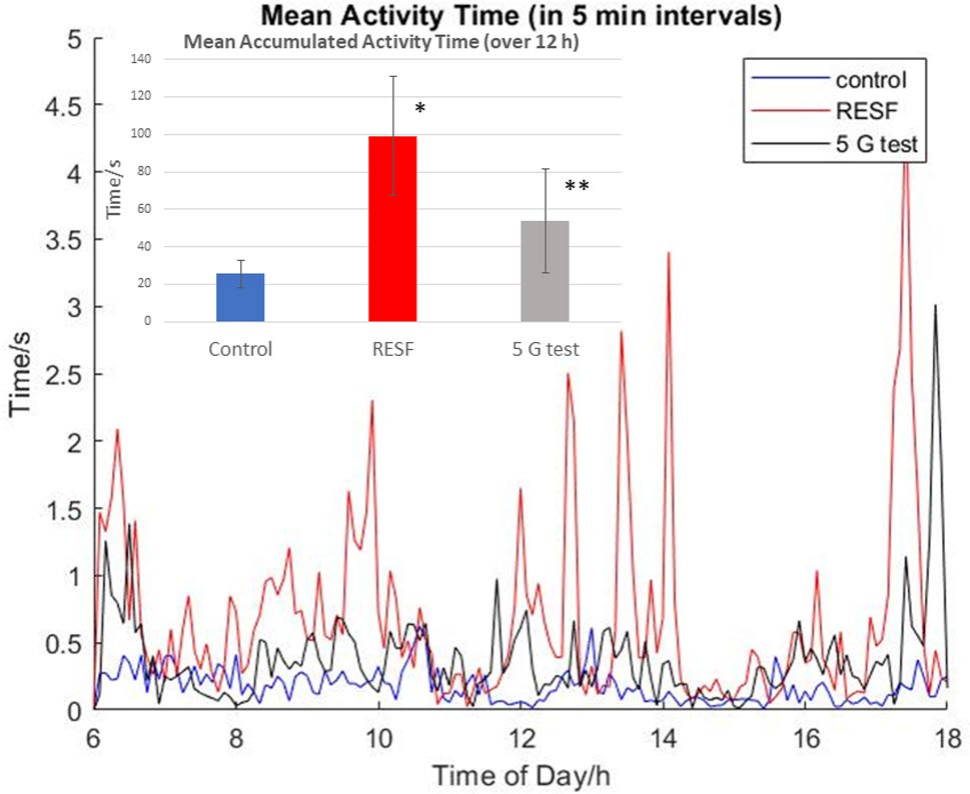
Mean activity time of the American cockroach (N = 29 for controls and RESF, N = 16 for 5 G test). The main plot shows the time a cockroach spends in motion (translating or rotating), averaged over all measured cockroaches. The curves are obtained by connecting points showing the mean activity time in the preceding 5 min time interval. The inset shows the mean accumulated activity time, i.e. the sum of the mean activity times over 12 h interval of the experiment. In the control runs the geomagnetic field is not modified, RESF stands for test runs with rotated Earth-strength field and 5 G test denotes experimental runs with horizontal compotent of the geomagnetic field rotated and its magnitude increased to 5 Gauss. The error bar is the standard error. *p value = 0.028 (t test against controls), **p value = 0.222 (t test against controls).

From the obtained videos, cockroach position and angle in the horizontal plane was extracted for every frame and used to compute the activity time, defined as the time the cockroach spends in motion—translating or rotating (see “Data analysis” in “Methods” section). Figure 2 summarizes the obtained results. A statistically significant difference in the mean accumulated activity time (over the 12 h duration of the experiment) is observed between the RESF and control conditions (t test, p value = 0.028), but not between the 5 G tests and the controls (t test, p value = 0.222).

## Discussion

The main plot in Fig. 2 is composed of points showing the mean activity time in the preceding 5 min interval. The mean activity time is the amount of time a cockroach spends in motion, averaged over all measured cockroaches. Low activity times indicate that cockroaches tend to be stationary (note that the mean activity time in Fig. 2 is at most 100 s over a period of 12 h, suggesting the cockroach does not move in general). This is expected as cockroaches are nocturnal animals and our experiment monitors them during day hours. Accordingly, they are mostly sleeping, moving from time to time, which is reflected by the spikes in the plot. We observe that the mean accumulated activity time is increased in RESF test condition, as compared to the control runs. In the 5 G test condition, this effect is diminished. This confirms sensitivity of the American cockroach to the direction of external magnetic field observed by the group of Dr. Martin Vacha in Masaryk University (0.17 G horizontal component, inclination $69^\circ$) but with different geomagnetic field conditions (in Singapore we measured 0.35 G horizontal component, inclination $28^\circ$). Furthermore, it indicates that the sensing mechanism is tuned to external fields with magnitudes similar to the Earth’s field. It is to be expected that cockroaches are magnetically sensitive anywhere on Earth.

We now discuss two main candidate models of magnetoreception in the light of the obtained results—the magnetite model and the radical-pair model. They have been originally suggested for animal orientation or migration, but of course they are natural also for explanation of magnetosensitivity. We will focus on the physical part of these models and will not touch upon how the relevant sensing information is transduced to the brain, although from time to time we will mention relevant literature and its relation to the present study. Due to the variable strength of the magnetic field geographically and for simplicity in the theoretical models below we assume a horizontal geomagnetic field of 0.5 G.

### Magnetite model

The magnetite model supposes that magnetic materials inside the animal rotate as little compasses in an external magnetic field. These rotations are transduced to the nervous system and interpreted. The hypothesis gained popularity with the discovery that certain bacteria are capable of biomineralising magnetic grains (either magnetite Fe$_3$O$_4$^2,26,27^ or greigite Fe$_3$S$_4$^28^ or both^29^) via specialised organs. Other animals, including humans, can also mineralise magnetic particles^30^. In particular, magnetite particles with grain sizes in the range of 10–450 nm were extracted from several species of insects^3,31–33^. The smaller of such cube-like particles, up to 200 nm, are stable single-domain materials^34,35^. The bigger particles are hard to distinguish from external magnetite present, e.g., in soil^32^. Thus, most biogenic magnetites are thought to be single-domain particles. One also verifies plausibility of this model by noting that magnetic energy of the single domain grain of magnetite with radius 50 nm placed in the Earth’s magnetic field is almost 10 times larger than the thermal energy at room temperature. Furthermore, the cockroaches were observed to acquire magnetic moment in the presence of a strong magnetic field, confirming presence of magnetic materials in their bodies^22^. Due to observed long demagnetisation times Kong et al. estimated that a magnetite particle of radius $R = 50$ nm, saturation magnetisation $M_s=3 \times 10^5$ A/m and mass density $\rho = 4.049 \times 10^3$ kg/m$^{3}$ has to rotate in medium with high viscosity $\eta = 10^5$ Pa s^22^. We now demonstrate that such magnetic grains and their surroundings are disqualified by our data.

To this end, we first consider a simple simulation of non-interacting single domain magnetic grains and later argue that it qualitatively extends to more complex rotating magnetic moments (that can model chains of magnetic particles). We constrain our model to two dimensions and monitor the rotational motion only in the horizontal plane. To account for various possible initial orientations, each simulation begins with the magnetic moment at angle $\theta \in \{10^\circ , 20^\circ , \dots , 360^\circ \}$ from the direction of the geomagnetic north. This has the macroscopic effect of cancelling out the initial magnetisation contributions from each individual grain, agreeing with the experimental observation that cockroaches have no residual magnetisation^22^. In the presence of an external magnetic field, the moments gradually align toward the field. The rotational motion of the *i*th moment is described by Newton’s law:

1 $$\begin{aligned} I \ddot{\theta _i} = -f \dot{\theta _i}-\mu B \sin \theta _i, \end{aligned}$$

where $\theta _i$ is the angle between the external field and the *i*th magnetic moment, $I = \frac{2}{5}\rho V R^2$ is the moment of inertia of a ball, and $f = 8\pi \eta R^3$ the corresponding rotational friction coefficient. Note that we have ignored the thermal torque which will additionally misalign the particles and reduce magnetisation of the whole set. We have simulated the dynamics of the moments for 12 h, changing the direction of the external field every 5 min as in the experiment. In order to make quantitative comparisons between the different experimental conditions, we compute the “alignment” defined as:

2 $$\begin{aligned} M = \frac{| \sum _{i = 1}^n \vec m_i |}{n |m|}, \end{aligned}$$

where *n* is the total number of particles, $\vec m_i$ is the magnetic moment of the *i*th grain and |*m*| is its magnitude (we assume that all the magnetic moments have the same magnitude). *M* can be thought of as the ratio of magnetic moments aligned along the axis defined by the vectorial sum of all the moments. *M* equals 0 when the individual moments exactly cancel out and it is equal to 1 when all the magnetic moments are parallel.

**Figure 3:**
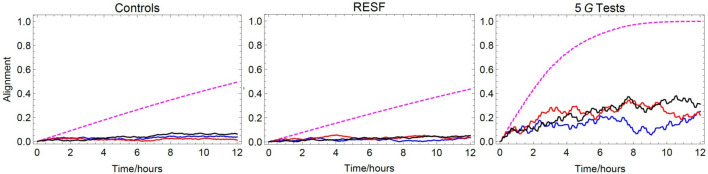
Alignment, as defined in Eq. (2), in the magnetite model (magnetite particle of radius 50 nm, viscosity $10^5$ Pa s). Alignment is a dimensionless and normalised quantity that captures the ratio of magnetic moments along the axis determined by their vectorial sum. The dashed line shows the alignment independent of cockroach orientation, while solid colorful lines include experimentally determined cockroach motion (for three typical insects).

The results of our simulations are presented in Fig. 3 and in the Supplementary Information. It turns out that in the presence of an Earth-strength magnetic field, a highly viscous environment leads to the alignment of only about $40 \%$ of all the magnetites after 12 h. In the 5 G field, the alignment is complete. One expects that the higher alignment is easier to sense by the animal. Under this assumption the model shows that 5 G field should be more disturbing to the insect than the Earth-strength field and accordingly the mean accumulated activity time should be at least as high. This is opposite to experimental findings.

Additionally, we ask if the motion of the cockroach is an attempt to maintain a certain preferred alignment. For this, let us denote the measured angle of the insect in the *j*th frame by $\theta _j^{\text {exp}}$. We now modify the angle of the magnetic grain at times $t_j = j \Delta t$, where $\Delta t$ is the time duration between the frames, by adding to them the cockroach motion, i.e. $\theta _j \rightarrow \theta _j +\theta _j^{\text {exp}}$. The simulations show that the motion does not lead to improved alignment. In fact, in the Earth-strength field, the motion inhibits *M* which stays below 0.05 level. Note that this holds for both control and test experiments, again in disagreement with experimental finding that cockroaches behave differently in these two conditions. We also verified that these conclusions stay the same if instead of adding the motion $\theta _j^{\text {exp}}$ we allow for a delay, i.e. add motion from the past, $\theta _{j-d}^{\text {exp}}$.

Presented simulations deal with a simple model that is already relevant to a number of transduction proposals, e.g. the membrane short model and torque detectors^13^ which rely on rotational motion of individual magnetic grains^36^. Rotating magnetites can even be further complemented by tinier magnetite nanoparticle clusters in the osmotic magnetometer models proposed in^37^. One could extend our model to more complicated magnetic structures or strongly interacting magnetites (such that they collectively behave as a larger rigid structure) by modifying magnetic moment, moment of inertia and rotational friction coefficient. In particular, rods are the shapes of special importance because they model chains of magnetic grains and have been considered as part of the transduction related to opening of ion channels^38^. We have run the simulations of rotations of magnetic rods (in SI) and observe qualitative agreement with the results in Fig. 3. In general, it is hard to envision any mechanistic model compatible with the diminishing activity for a larger aligning field if one expects that higher alignment translates to better sensing and higher activity. It is a generic property of all such models that stronger fields lead to a better alignment. For example, models with different viscosity values and different sizes of single-domain magnetites are also all excluded.

### Radical pair model

The radical-pair mechanism was first conceived in 1969 by Closs, Kaptein and Oosterhoff (CKO model)^39,40^ as an explanation of chemically induced dynamic nuclear polarization, which has ever since been an important technique in NMR spectroscopy^41,42^. It was later proposed by Schulten et al.^14^ to be involved in animal magnetoreception.

The Cryptochrome/Photolyase flavoprotein family are thought to be relevant to light-sensitive biological compasses^16^, hinting at the radical pair model. We base our discussion on the work by Solov’yov et al.^43^ who described creation of radical pairs and their dynamics in Cryptochrome-1 (Cry-1) of the plant Arabidopsis thaliana. While Cry-1 was absent in *Periplaneta americana*, Cryptochrome-2 was found to be present and also necessary for magnetic sensitivity^25^. The parameters below are taken from experiments on Photolyase in *E. coli* (hyperfine axes)^44^ and Cry-1 in Arabidopsis thaliana (transition rates)^45,46^. This is justified given that the radical pair mechanism is believed to be contained within the highly conserved FAD molecular domain^47^, common across the Cryptochrome/Photolyase family.

**Figure 4:**
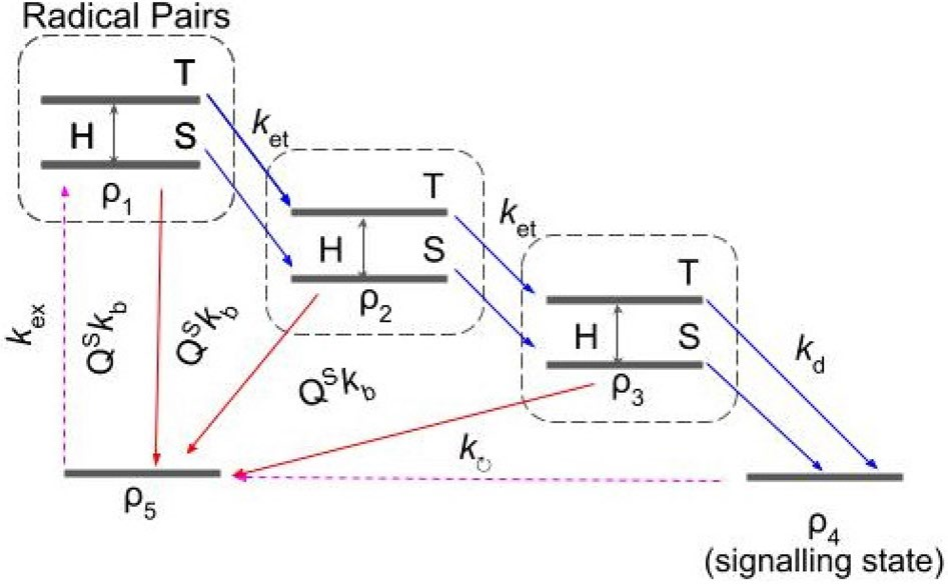
Effective model of the cryptochrome radical pair mechanism. The first radical pair is created in the state $\rho _1$ by an excitation of the precursor molecule $\rho _5$. Electron hopping gives rise to other pairs, $\rho _2$ and $\rho _3$. Their spin dynamics, coherent evolution between the singlet (S) and triplet (T) states, depends on the external magnetic field and they can recombine to the precursor only if they are in the singlet state. This selective recombination is represented by the singlet projectors $Q^S$ in front of the back-transfer rates, $k_b$, and modifies the yield of the signalling state $\rho _4$, perceived by the animal. Variants of the model with solid transitions have been studied previously, see e.g.^43,48,49^. We also consider the model with additional dashed arrows, which close the dynamics of the whole system.

The radical-pair reaction pathway given in^43^ can be compressed to essential steps given in Fig. 4. The model begins from the photo-excitation and protonation of FAD (FAD $$\rightarrow $$ FAD$$^* \rightarrow $$ (FADH$$^+)^*$$). An electron from a nearby Trp-400 can fill this hole to create the first radical pair, FADH + Trp-$$400^+$$, which we denote as $$\rho _1$$. The now unpaired electrons on each half of the radical pair initialise in the singlet state. Due to the proximity of electron-accepting molecules, one of the electrons may hop around, giving rise to a chain of radical pairs $$\rho _1 \rightarrow \rho _2 \rightarrow \rho _3$$ connected with electron transfer rate $$k_{\mathrm {et}}$$. These three radical pairs correspond to FADH + Trp-$$400^+$$, FADH + Trp-$$377^+$$ and FADH + Trp-$$324^+$$, respectively. The rate $$k_{\mathrm {et}}$$ has been measured to be on the order of $$10^8$$ Hz^43^. There is also a possibility of reverse electron transfer, but its rate is estimated to be two orders of magnitude smaller and therefore we ignore it. The whole system evolves in the external magnetic field $$\vec {B}$$ with each unpaired electron coupled to a nearby distinct nuclei, so that the total Hamiltonian is the sum of the following terms:

3 $$\begin{aligned} H_j= 2 \mu _B \vec {B} \cdot \vec {S}_j + \mu _B \sum _i \vec I_i \cdot (\overleftrightarrow {A}_{ij}^{\text {iso}} + \overleftrightarrow {A}_{ij}^{\text {aniso}}) \cdot \vec {S}_j, \end{aligned}$$

where the subscript $i=1,2$ refers to the nuclei, $j=1,2$ to the electrons, and $\mu _B$ is the Bohr magneton. The first term is the Zeeman interaction of the electron spin $\vec S_j$ with the external magnetic field, with the g-factor of 2. The second term is the hyperfine interaction with nuclear spin $\vec I_i$ that couples to the electronic spin with diagonal hyperfine tensor $\overleftrightarrow {A}_{ij}^{\text {iso}}$ and anisotropic tensor $\overleftrightarrow {A}_{ij}^{\text {aniso}}$. The radical pairs have a fixed orientation within a larger photolyase with a molecular axis that is the reference axis in the model. A full description of the geometry can be found in Ref.^44^. The relevant tensor elements used in our model have been experimentally measured^44,50–52^ and we summarise the values used in our simulations in SI. Due to this interaction, the pair coherently oscillates between singlet and triplet states and can selectively recombine to the precursor of the radical pairs $\rho _5$, with the estimated rate $k_b = 10^7$ Hz, only if the pair is in the singlet state. This is the key reason for the reaction pathway to be magnetically sensitive since this interconversion is controlled by the strength and direction of $\vec {B}$. Finally, the last pair in the chain, $\rho _3$, can decay to the signalling state $\rho _4$, with the measured rate $k_d = 3.3 \times 10^6$ Hz. The amount of $\rho _4$ is assumed to be perceived by the animal. All the transfers are incoherent processes and are described with the help of the rate equations. The final figure of merit is the yield of the signalling state $\Phi$ being the probability of finding the system in $\rho _4$ after a long time.

**Figure 5:**
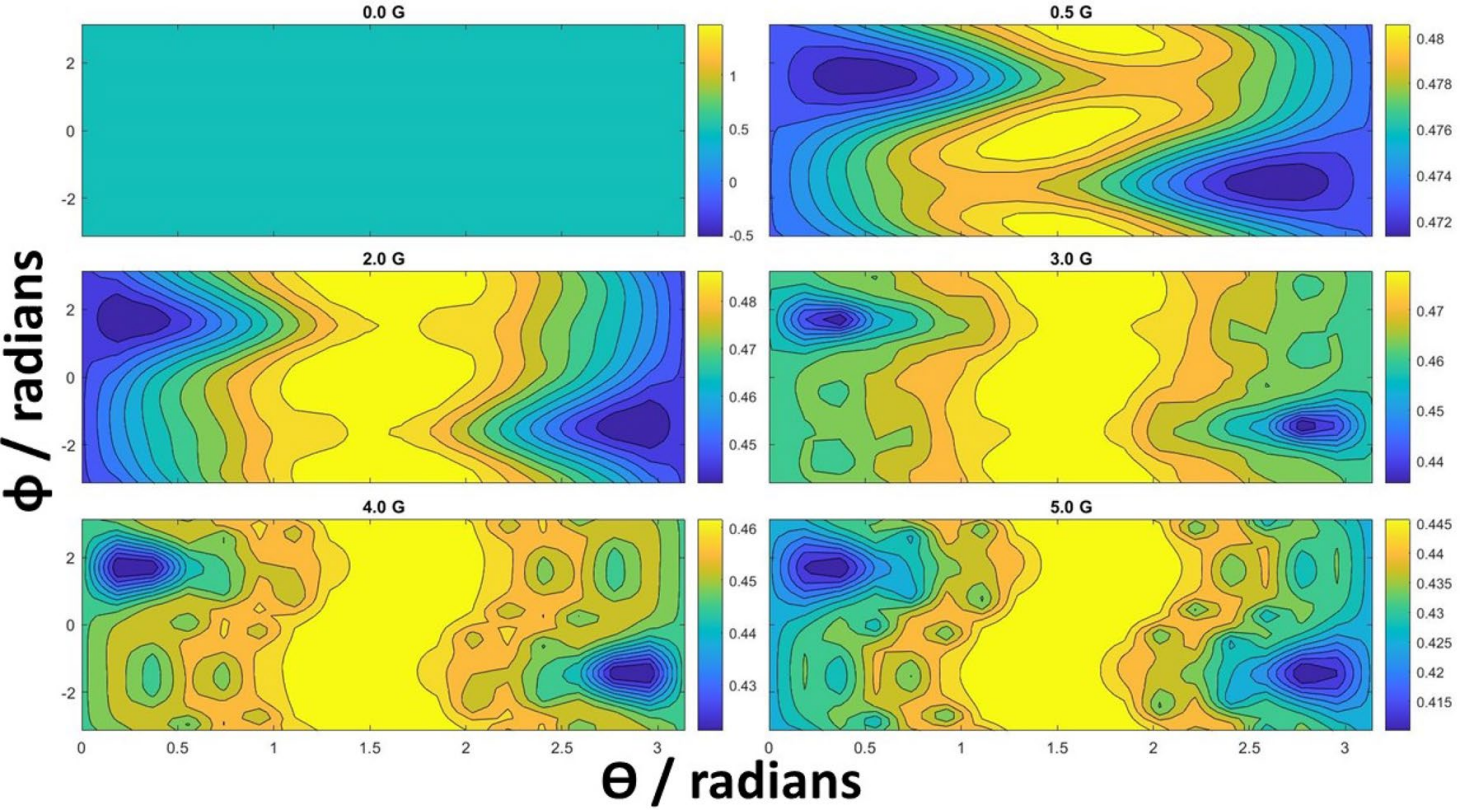
Signalling yield as a function of external magnetic field in the radical pair model with solid arrows in Fig. 4. The field is parameterised in spherical coordinates $\vec {B}=(B_0 \sin \theta \cos \phi , B_0 \sin \theta \sin \phi , B_0 \cos \theta )$, with the photolyase molecular axis lying along the z-axis. The evolution in the magnetic field is governed by the Hamiltonian given in Eq. (3), using the hyperfine tensors measured via EPR and ENDOR techniques^44,50^, given in the Supplementary Information. The more complex structures visible for stronger external fields are the effect of comparable hyperfine interactions (in the range 4–16 G) and external fields.

In the Supplement we describe validation of our numerics with analytical estimates and compare our simulations to those of Ref.^43^. Note that the timescale of the radical pair dynamics is in the order of microseconds, which is significantly faster than our 5 min periodically rotated field. This means that we do not need to take into account the field rotations as we have done for the magnetite model. Figure 5 shows $\Phi$ as a function of spherical polar and azimuthal angles of the external magnetic field relative to the photolyase molecular axis, for various magnitudes of the field. There is a variation of the yield with the direction of the external field for all magnitudes. In particular, for the Earth-strength field of 0.5 G, the contrast $\Phi _{\max } - \Phi _{\min }$, is around 0.008 and yield is about 0.48, whereas in the 5 G field, we have a contrast of 0.04 and a yield smaller than 0.44. In principle, the result of our experiment could be explained within this model by adding an assumption that the sensing requires the signalling yield above a threshold value of about 0.45, and that changes in the yield on the order of one in a thousand are perceivable by the animal.

**Figure 6:**
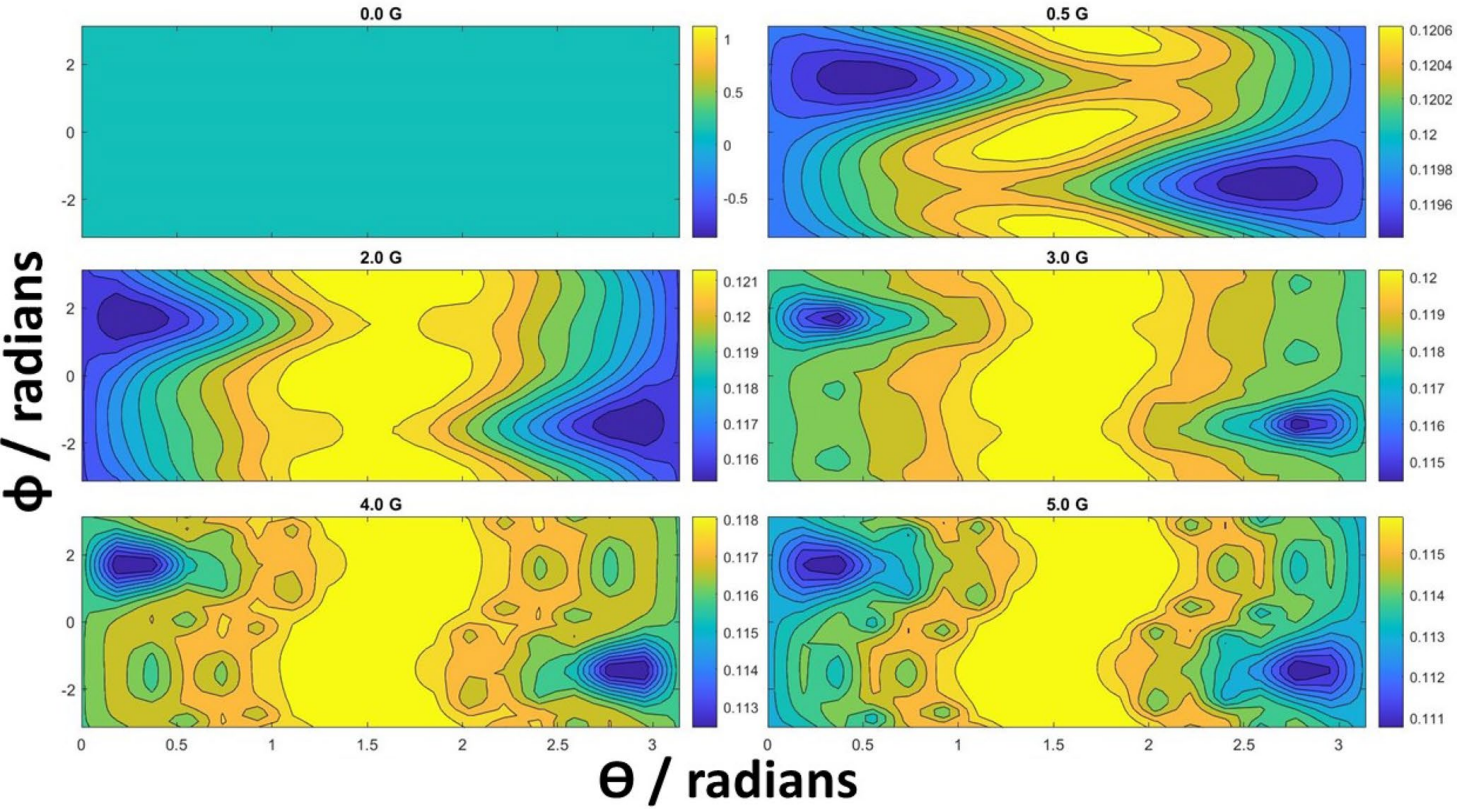
Signalling yield as a function of external magnetic field in the radical pair model with closed dynamics, i.e. with additional dashed magenta arrows in Fig. 4. Evolution under the magnetic field is governed by the Hamiltonian given in Eq. (3). In this particular plot, we have set $k_{\mathrm {ex}}=5 \times 10^6$ Hz and $k_{\circlearrowright } = 10^7$ Hz. The model preserves compass features, but the yield and variations are diminished.

The dynamics of this model depends on the hyperfine tensor elements and, to a lesser extent, on the decay constants $k_b$, $k_d$, and $k_{\mathrm {et}}$. The latter affect incoherent transfer of populations and control the numerical value of the signalling yield without drastically affecting the profile. From Eq. (3), the anisotropic terms coupling nuclei with electrons give rise to the non-trivial angular dependence. For example, using the numbers in Table 1 of the Supplement, the first electron experiences a strong coupling of almost 10 G to the nucleus along the axes presented in the third row. This corresponds to angular components of about $\theta =0.3$ rad and $\phi =1.4$ rad, which we observe as minima in all the plots in Figs. 5 and 6.

The model that was just described begins with the formation of radical pair and ends with the signalling state as a sink. Many natural processes are closed and we now ask about the possibility of a self-sustaining biocompass that can recycle the signalling state. For this, we add to the presented radical-pair model two transitions (denoted with dashed magenta lines in Fig. 4) that close the loop of the process. The transition $\rho _5 \rightarrow \rho _1$ occurs with the rate $k_{\text {ex}}$ and captures the rate of creation of the first radical pair, typically as a result of illumination with sunlight. The transition $\rho _4 \rightarrow \rho _5$ occurs with the rate $k_{\circlearrowright }$ and describes conversion of the signalling chemical to the initial precursor molecule. Numerical simulations show that the compass retains its functionality only if both of these new rates are similar. If $k_{\circlearrowright } \gg k_{\text {ex}}$ the system accumulates in the precursor state $\rho _5$ and if $k_{\circlearrowright } \ll k_{\text {ex}}$ the steady state is the signalling state $\rho _4$ independently of the external magnetic field. Figure 6 shows the results for exemplary set of $k_{\text {ex}} = 5 \times 10^6$ Hz and $k_{\circlearrowright } = 10^7$ Hz. The variations of the yield are qualitatively similar to those in Fig. 5 but quantitatively the yield is about ten times smaller and its variations are also correspondingly smaller. This shows that closing the dynamics has important consequences and makes the radical pair hypothesis more difficult to sustain.

## Conclusions

In conclusion, we conducted behavioural experiments on American cockroaches (*Periplaneta americana*) that confirm their sensitivity to directional changes of Earth-strength magnetic fields of about 0.5 G. The sensitivity is revealed by increased activity of insects during daytime. Furthermore, the data from experiments in 5 G periodically rotated fields show diminished activity, indicating adaptation of the sensing mechanism to the Earth’s magnetic field. A similar observation has been made for European robins exposed to fields 2 times stronger than the Earth’s field^53^, as well as fields 10 times stronger for the honeybee *Schwaziana quadripunctata*^54^. We performed numerical analysis of the usual theoretical candidates of magnetoreception, the magnetite and radical-pair models, in light of the obtained results. In our analysis of the magnetite model we take the sizes of the single-domain magnetic grains that were extracted from insects. If one assumes that better alignment of the grains to external field translates to better sensing ability and higher insect activity, these models cannot explain our experimental observations. The radical-pair model, with the parameters presently available from the experiments on Photolyase and Cry-1, can only explain the sensitivity to the Earth-strength field and its diminishing effect in the stronger field, if we assume that contrast in the chemical yield on the order of one in a thousand is perceived by the animal, and additionally that this only happens if the yield is above a certain threshold (attained in 0.5 G but not in 5 G field).

## Methods

### Experimental procedure

The experiments were conducted in Singapore (measured horizontal component of the geomagnetic field in our laboratory was 0.35 G and inclination angle was $28^\circ$). All cockroaches were kept in separate transparent insectaria with unlimited water, a diet consisting of cat food pallets and photo-period of 12 light (6 am to 6 pm): 12 dark (6 pm to 6 am) hours. A day before the experiment, at 6 pm, a single insectarium was placed in a $4\,^{\circ }$C environment in order to immobilise the insect, which was then moved to a Petri dish (15 cm diameter) with circumference covered with white slip. To minimise visual cues, the Petri dish was placed in a box with the inside surfaces covered by white paper, except two opposite facing sides with white translucent films to allow external lights, 2 $\times$ 600 lumen, 10W LED bulbs (Ikea LED1424G10), for uniform illumination inside the box. A small aperture was made on the overhead for the camera configured with a capture rate of 30 frames per second. The box was installed on a stage levelled in the middle zone (where the generated magnetic field is most uniform) of a Merritt four square coil^55^.

The cockroach was left overnight in an isolated room in order to acclimatise with the new environment. The experiment is automated here on, with the room sealed till 6 pm on the next day; recording, lighting and magnetic conditions automatically commence at 6 am the next day. In the RESF test runs the magnetic field was alternating between the natural geomagnetic field in Singapore and a field with the horizontal component of the geomagnetic field rotated clockwise by $60\pm 5^\circ$, without a change in the vertical component. This rotation was realised by switching on the Merritt coil (side dimension 1.2 m), levelled horizontally and arranged at $120\pm 5^\circ$ from the geomagnetic north. The magnitude and inclination angle of the rotated field was the same as for the geomagnetic field. A Honeywell HMR2300 smart digital magnetometer was used to verify the magnetic field in every run. In the 5 G test condition the magnetic field was alternating between the natural geomagnetic field in Singapore and a field with the horizontal component of the geomagnetic field rotated clockwise by $60\pm 5^\circ$ and the magnitude of the horizontal component increased to 5 G, without a change in the vertical component. For this we used a smaller Merritt coil (side dimension 0.3 m) arranged at suitable angle. See Supplementary Information for illustration. The remaining procedures were the same. The same procedures were also followed in the control runs, except that the coils were not switched on at all.

### Data analysis

The experiments output video recordings of cockroach motion in the Petri dishes. In order to obtain numerical parameters easily comparable between tests and control runs, the videos were processed as follows. The self-written tracking software (C++) identified the cockroach in every frame, fitted an ellipse to it, and stored in a text file timestamp, coordinates of the center of the ellipse and angle of the main axis. From these values we computed activity time by summing up time intervals between the frames (33.3 ms) in which the center location changed by more than 3 mm or the angle changed by more than 8°. These thresholds are arbitrary and changing them does not significantly alter the profile in Fig. 2.

## Supplementary Information

Supplementary Information 1.

## Data Availability

The processed data should be available now at: https://osf.io/zk9d6/
