# Supplementary material for: Numerical tests of magnetoreception models assisted with behavioral experiments on American cockroaches
Source: Sci Rep. 2021 Jun 9;11:12221. doi: 10.1038/s41598-021-91815-x (PMC8190300; doi:10.1038/s41598-021-91815-x)
Supplement: Supplementary file 1 — Supplementary Information 1. [file 41598_2021_91815_MOESM1_ESM.pdf]

Numerical tests of magnetoreception models assisted with behavioral experiments on American cockroaches — Supplementary Information

Kai Sheng Lee[1]**, Rainer Dumke**[1,2]**, and Tomasz Paterek**[1,3]

[1]School of Physical and Mathematical Sciences, Nanyang Technological University, 637371 Singapore, Singapore
[2]Centre for Quantum Technologies, National University of Singapore, 117543 Singapore, Singapore
[3]Institute of Theoretical Physics and Astrophysics, Faculty of Mathematics, Physics, and Informatics, University of Gdańsk, 80-308 Gdańsk, Poland

1 Alignment in the magnetite model

Here we show angular histograms giving further details about the alignment in the studied model, as well as for magnetites rods. The results of the simulations are shown in Fig. 1 and 2. The magnetites align toward the geomagnetic field (0°) as expected but there are still a significant number of particles not yet aligned. A similar outcome is observed in the RESF, except that the magnetites align toward the midpoint between the rotated field and geomagnetic field (30°). Due to the switching frequency being much smaller than the alignment timescale, the magnetites effectively experience the averaged magnetic field. In the 5 G test condition, the magnetites align much faster and eventually all 36 particles are aligned between 40° to 60°.

In our treatment of magnetite rods (length, $l = 150$ nm, diameter, $d = 107$ nm), we modified moment of inertia to that for a rod rotating along its center, $I = \frac{1}{12}mh^2$, and total dipole moment to match the dimensions. The rotational friction is taken to be that of an ellipsoid with semimajor axis, l, and semiminor axis, d, giving $f = 16\pi n l^3/(3(2\log(2l/d)-1))$ as from[1]. The obtained alignment is qualitatively the same as with magnetite spheres (plots below). This is understandable given that the leading terms in I and f are the "size" terms (length for rods and radius for spheres).

2 Hyperfine tensors

In our simulations of the radical pair model we used the hyperfine tensors as shown in Tab. 1, which have been measured using EPR and ENDOR techniques (see compilation in Ref.[2]). Note that these values are different from those used in Ref.[3].

First electron interacts with one nucleus				
A_{11}^{iso} [G]	A_{11}^{aniso} [G]	hyperfine axes		
3.93	-4.98	0.4380	0.8655	-0.2432
	-4.92	0.8981	-0.4097	0.1595
	9.89	-0.0384	0.2883	0.9568
**Second electron interacts with two nuclei**				
A_{12}^{iso} [G]	A_{12}^{aniso} [G]	hyperfine axes		
13.6	0	1	0	0
	0	0	1	0
	0	0	0	1
A_{22}^{iso} [G]	A_{22}^{aniso} [G]	hyperfine axes		
-4	-2.3	-0.984	0.180	0
	3.5	0.180	0.984	0
	-1.2	0	0	1

Table 1. Hyperfine tensors used in our simulations of the radical pair model.

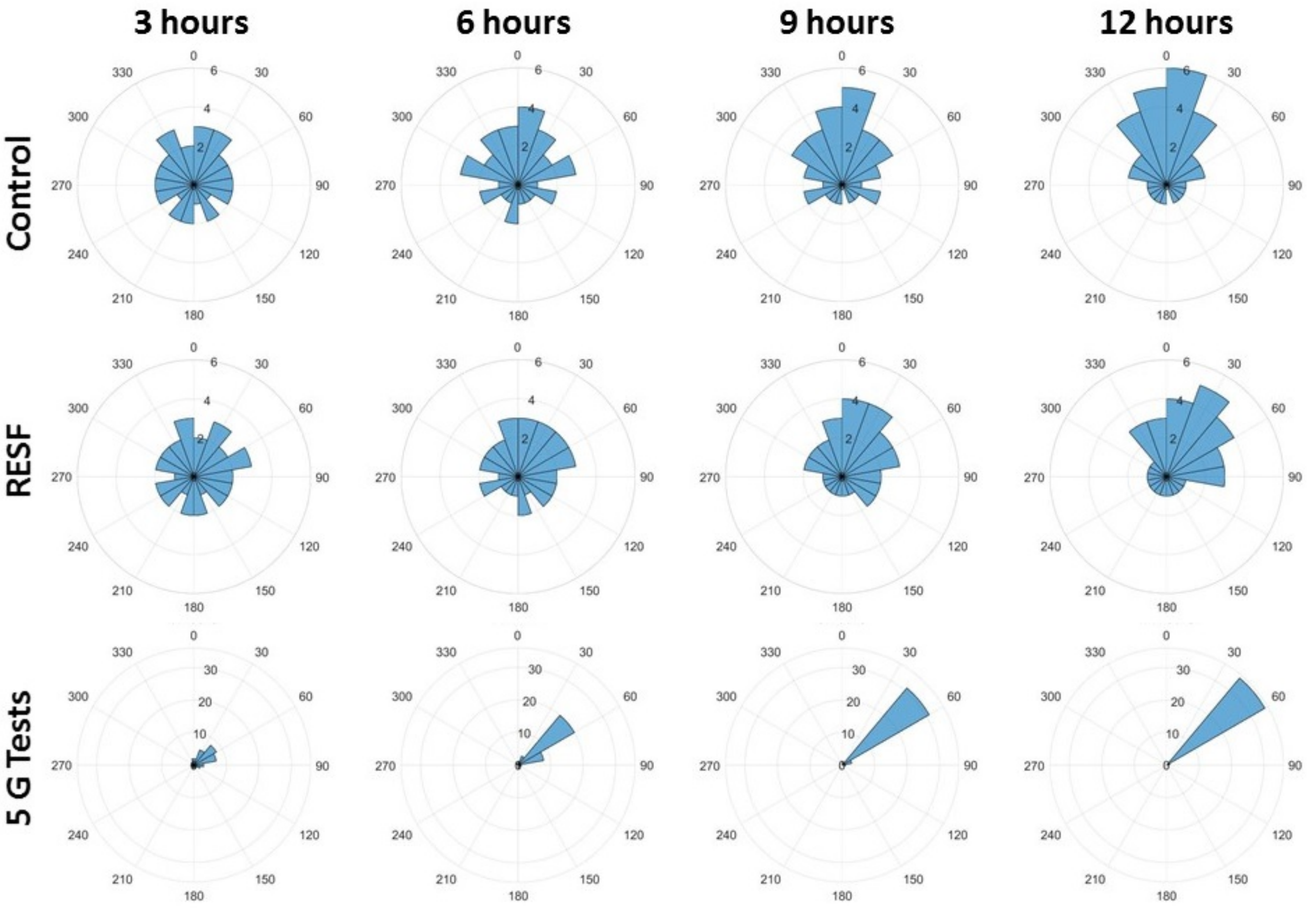

Figure 1. The angular histograms of 36 magnetic moments studied in the main text (magnetite spheres of radius 50 nm, viscosity 10^5 Pa sec). We have run 36 simulations, each starting with a single magnetic moment at angle $\theta = \{10^\circ, 20^\circ, \ldots, 360^\circ\}$. The panels from left to right show the number of magnetic moments at an angle from magnetic north at 0° (angular resolution, i.e. bin size is 20 degrees) and at different times as indicated (each consecutive panel three hours later). The first row is the model of the control runs (no changes of magnetic field). The second row models test runs in rotated Earth-strength field (RESF) and shows partial alignment to angle of 30°, i.e. in between rotated and geomagnetic field. The third row models the 5 G test condition and shows very good alignment after 9 hours to the angle of almost 60° (compatible with the fact that rotated field is twelve times stronger than the geomagnetic field). Note that in keeping a consistent scale for comparison on the last row, some of the bars in the two bottom left row panels (5 G Tests for 3 hours and 6 hours) are obscured.

3 Validation of numerics

We describe here in more detail how we have verified accuracy of our numerics as certain obtained results are different from those in the literature. In particular, Ref.[3] computes the variation in time of the probability that the radical pair ρ_3 is in the singlet / triplet state. Both are found to be at most 0.05 within the first 500 ns of evolution (Fig. 10 of that reference). We now give analytical estimates of these probabilities which turn out to be an order of magnitude higher and provide the following intuitive explanation why higher values should be expected. Recall that the electron transfer rate is an order of magnitude faster than the recombination rates and two orders of magnitude faster than the decay rate to the signalling state. Therefore, all the dynamics in the chain $\rho_1 \to \rho_2 \to \rho_3$ is fast and one expects non-negligible portion of pairs in the state ρ_3.

In order to place the analytical bounds on the probability that the system is in ρ_3 (independently of whether it is the singlet or triplet state) we note that setting the recombination rate to zero gives the upper bound to the population in ρ_3, whereas allowing recombination independent of the spin state provides the lower bound on the population. Since in both cases the system looses spin dependence it is governed by the following set of rate equations, which can be read out from the reaction

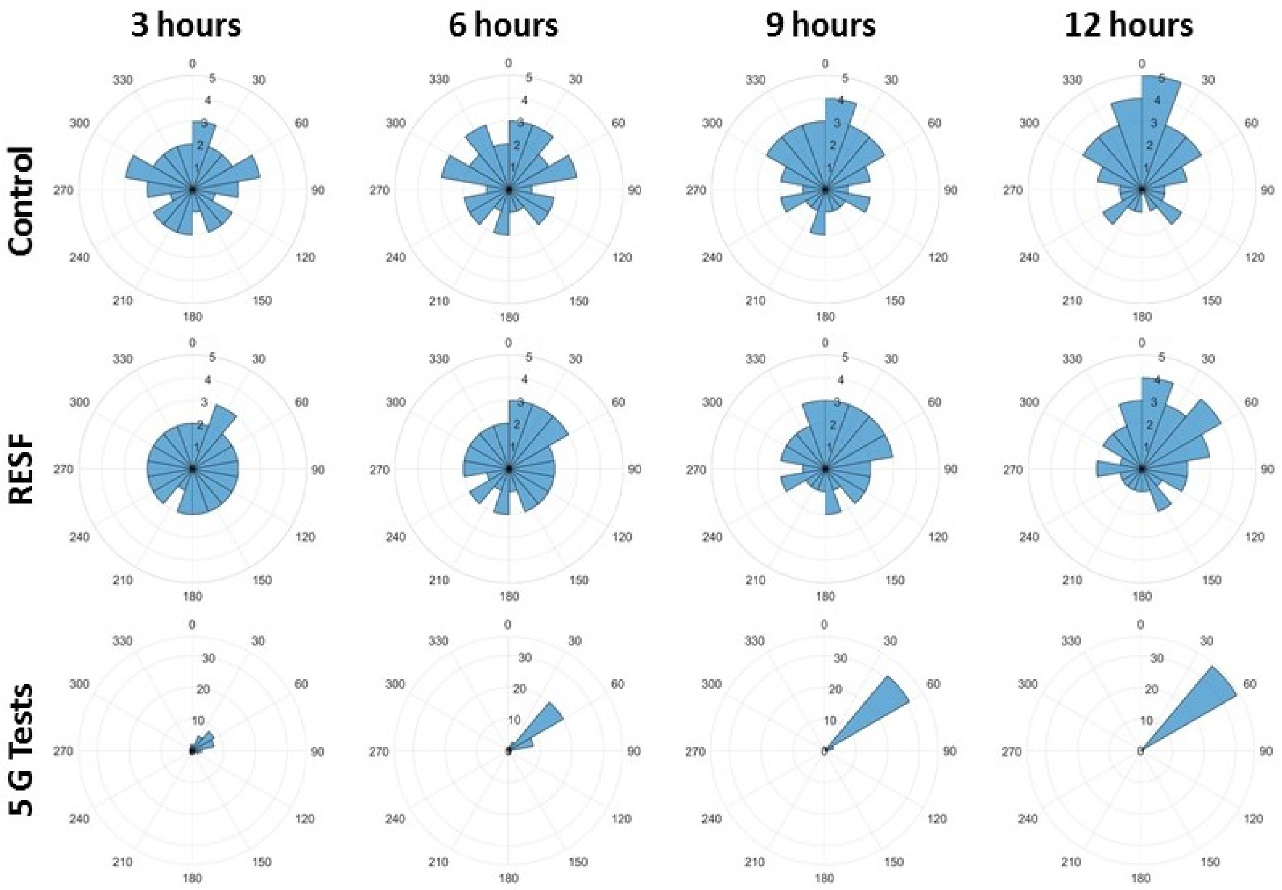

Figure 2. The angular histograms of 36 magnetite moments (cylindrical rods of length 150 nm and diameter 107 nm, viscosity 10^5 Pa sec). The results are qualitatively similar to magnetite spheres.

scheme in Fig. 4 of the main text:

$$\begin{aligned} \dot{p}_1 &= (-k_{\text{et}} - v k_b)\, p_1, \\ \dot{p}_2 &= (-k_{\text{et}} - v k_b)\, p_2 + k_{\text{et}}\, p_1, \\ \dot{p}_3 &= (-k_d - v k_b)\, p_3 + k_{\text{et}}\, p_2, \end{aligned} \tag{1}$$

where p_j is the population in the jth radical pair and $v = 0, 1$ turns on and off the possibility of recombination. This set of equations admits analytical solution:

$$\begin{aligned} p_1 &= \exp[(-v k_b - k_{\text{et}})t], \\ p_2 &= k_{\text{et}}\, t\, p_1, \\ p_3 &= \frac{k_{\text{et}}^2}{(k_{\text{et}} - k_d)^2} \left[\exp\left((-v k_b - k_d)t\right) - p_1\right] - \frac{k_{\text{et}}}{k_{\text{et}} - k_d}\, p_2. \end{aligned} \tag{2}$$

The corresponding limiting curves (for $v = 0$ and $v = 1$) are plotted in Fig. 3 (dashed-dotted lines). The plot confirms that the populations p_1 and p_2 quickly decay to zero and shows that the number of radical pairs ρ_3 lies well within the obtained boundaries. Note that the results in Ref.[3] are below the analytical lower bound obtained here.

4 Magnetic field conditions

The magnetic fields used in different experimental conditions are shown in Fig 4. Note that the vertical component is always the same.

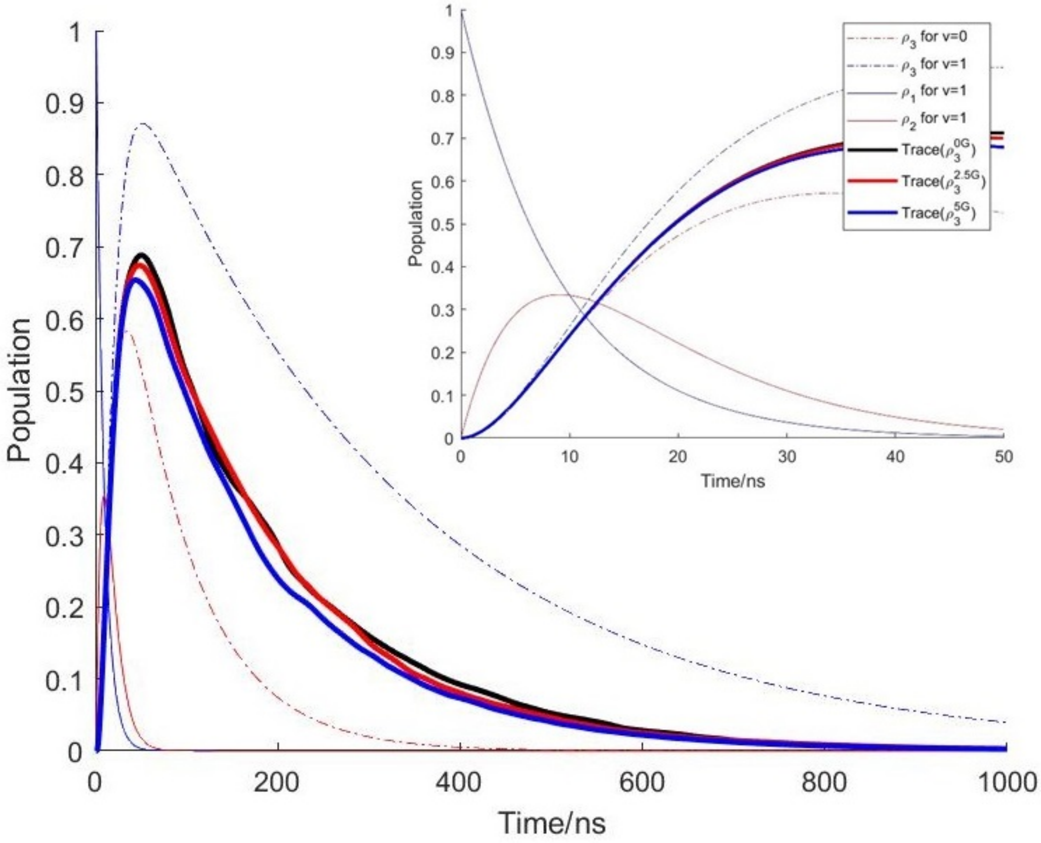

Figure 3. Population dynamics of the radical pairs. The solid curves are computed with the rates and the hyperfine tensors as in Ref.[3]. The population of the first two radical pairs is transferred to the third pair within 50 ns. The inset is a close-up for the timescale up to 50 ns. The population of the third pair is plotted in thick lines for various strengths of external magnetic field. All of them lie within the obtained analytical bounds shown by dashed-dotted lines.

References

1. Yorke, E. D. A possible magnetic transducer in birds. J. Theor. Biol. **77**, 101–105, DOI: 10.1016/0022-5193(79)90140-1 (1979).
2. Cintolesi, F., Ritz, T., Kay, C., Timmel, C. & Hore, P. Anisotropic recombination of an immobilized photoinduced radical pair in a 50-μT magnetic field: a model avian photomagnetoreceptor. Chem. Phys. **294**, 385–399 (2003).
3. Solov'yov, I. A., Chandler, D. E. & Schulten, K. Magnetic field effects in arabidopsis thaliana cryptochrome-1. Biophys. J. **92**, 2711–2726 (2007).

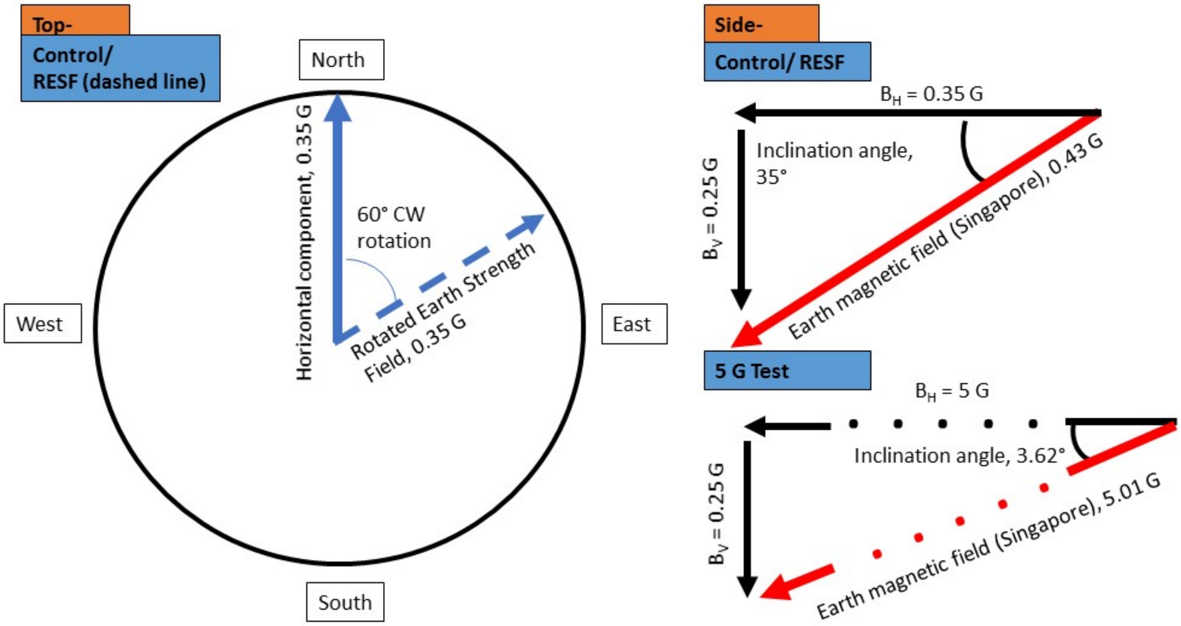

Figure 4. Magnetic conditions. Left panel: top view of the rotated Earth-strength field (RESF) condition. The solid line gives the measured horizontal component of the geomagnetic field. Top right panel: Side view of the RESF condition. Bottom right panel: side view of the 5 G test condition after rotation of the horizontal component and increment of its magnitude to 5 Gauss. We used dots to indicate that this vector is much longer than the corresponding one in the top right panel.
